# 非小细胞肺癌细胞株*TGFBR3*基因缺陷表达及其分子机制研究

**DOI:** 10.3779/j.issn.1009-3419.2010.05.14

**Published:** 2010-05-20

**Authors:** 谢芳 蒋, 仍允 刘, 哲 雷, 嘉琮 尤, 清华 周, 洪涛 张

**Affiliations:** 1 215123 苏州，苏州大学癌症分子遗传学实验室 Soochow University Laboratory of Cancer Molecular Genetics, Suzhou 215123, China; 2 300052 天津，天津医科大学总医院，天津市肺癌研究所，天津市肺癌转移与肿瘤微环境重点实验室 Tianjin Key Laboratory of Lung Cancer Metastasis and Tumor Microenvironment, Tianjin Lung Cancer Institute, Tianjin Medical University General Hospital, Tianjin 300052, China

**Keywords:** 非小细胞肺癌细胞株, TGFBR3, 基因突变, DNA甲基化, 转移, Non-small cell lung cancer cell lines, TGFBR3, Gene mutation, DNA methylation, Metastasis

## Abstract

**背景与目的:**

国外有研究表明，非小细胞肺癌（non-small cell lung cancer, NSCLC）中转化生长因子受体Ⅲ（TGFBR3）存在缺陷表达，但是分子机制尚未明确。本研究以正常支气管上皮细胞（human bronchial epithelial cell, HBEpiC）为对照，分析NSCLC细胞株中*TGFBR3*基因的表达情况，并探讨*TGFBR3*基因表达失活的分子机制。

**方法:**

采用Western blot检测HBEpiC和NSCLC细胞株中TGFBR3的表达情况并做相对定量分析；采用DNA直接测序检测TGFBR3基因启动子基本转录元件区的突变情况；应用亚硫酸氢钠处理后测序法（bisulfite sequence-PCR, BSP）检测TGFBR3基因启动子区甲基化状态。

**结果:**

NSCLC细胞株中TGFBR3表达水平明显低于HBEpiC；高转移细胞株95D明显低于非转移细胞株LTEP-α-2、A549、NCI-H460；HBEpiC与NSCLC细胞株中*TGFBR3*基因近端启动子区-165到-75区域无遗传突变，且未见甲基化，远端启动子区-314到-199区域均为高甲基化。

**结论:**

*TGFBR3*基因在NSCLC细胞株中表达下调，在高转移细胞株95D中尤其明显，提示该基因的表达缺陷对NSCLC发生发展起重要作用，可能与NSCLC的侵袭和转移相关；然而，*TGFBR3*基因启动子区重要转录元件区域的甲基化状态并不是导致*TGFBR3*基因表达下调的主要原因。

## Abstract (en)

**Background and objective:**

It has been reported that defective expression of TGFBR3 was found in non-small cell lung cancer (NSCLC). However, its molecular mechanisms remain unclear. The aim of this study is to investigate expression of TGFBR3 in NSCLC cell lines and normal human bronchial epithelial cell (HBEpiC), and to explore potential molecular mechanisms underlying inactivation of *TGFBR3* gene.

**Methods:**

Western blot was performed to determine the expression of TGFBR3 in HBEpiC and NSCLC cell lines. Automatic image analysis was carried out to estimate relative expression of TGFBR3 protein. We screened for mutation of the promoter region of *TGFBR3* gene using DNA direct sequencing. Bisulfite-sodium modification sequencing was used to detect the methylation status of *TGFBR3* promoter.

**Results:**

TGFBR3 protein level was abnormally reduced in NSCLC cell lines as compared with HBEpiC. There was significant difference in TGFBR3 expression between the highly metastatic cell line 95D and non-metastatic cell lines, including LTEP-α-2, A549 and NCI-H460. No mutation and methylation was found in upstream sites -165 to -75 of the proximal promoter of *TGFBR3* in HBEpiC and NSCLC cell lines. Hypermethylation was shown in upstream sites -314 to -199 of the distal promoter of *TGFBR3* in HBEpiC and NSCLC cell lines.

**Conclusion:**

Reduced expression of TGFBR3 was observed in NSCLC cell lines, especially in 95D, suggesting that TGFBR3 might play an important role in development and progression of NSCLC and correlate with NSCLC invasion and migration. The methylation event occurring at *TGFBR3* promoter is not a major cause for reduction of TGFBR3 expression.

目前所知不少生长因子、调节肽及其受体参与了癌症进程的调控。TGF-β信号转导通路蛋白的变异与肿瘤的关系是现今研究的热点之一。在肿瘤发生进程中TGF-β信号通路常表现出二元性^[1]^。肿瘤发生早期TGF-β可能作为肿瘤抑制因子，通过诱导细胞程序性死亡和细胞周期停滞从而抑制上皮细胞生长，但肿瘤恶化后TGF-β高表达，肿瘤细胞失去了对TGF-β信号的应答能力，肿瘤细胞自分泌的TGF-β通过增加透明质酸酶、纤维素酶的活性，引起基膜降解，提高肿瘤运行能力，促进肿瘤细胞转移，同时抑制周围正常细胞的增殖，影响淋巴细胞对肿瘤细胞的免疫作用，从而为自身癌细胞的生长筛选一个良好的内环境，发挥着促癌效应^[2]^。TGF-β信号通路通过调控血管发生、细胞增殖和分化，在肺癌发生发展过程中发挥着重要作用，大多数肺癌对TGF-β介导的抑制效应有耐受性，但其机制尚不清楚。TGF-β信号通路中的一种组分包括TGFBR2、smad2、smad4等的变异或缺失先前曾有研究^[3]^报道，然而这类变异在非小细胞肺癌（nonsmall cell lung cancer, NSCLC）中并不常见。Smad2和Smad4在肺癌中的变异只有5%-10%，大多数NSCLC中TGFBR1和TGFBR2均有表达^[4-6]^，而作为TGF-β信号通路中辅助受体的*TGFBR3*基因在NSCLC组织中常发生mRNA和蛋白水平上的表达缺失或下调^[7]^。

*TGFBR3*基因有两个选择性的启动子区（定义为proximal promoter和distal promoter）^[8]^，蛋白产物由851个氨基酸构成，是细胞内含量最丰富的一种类型蛋白多糖，又称betaglycan。它的表达与癌症的关系非常密切，在多种肿瘤中均有抑制癌细胞转移和侵袭作用，可调控多种肿瘤的进程^[9-11]^。TGFBR3在金属蛋白酶作用下进行溶蛋白性裂解，胞外功能区脱落后产生的可溶性sTGFBR3可以竞争性结合肿瘤自分泌的TGF-β，通过阻断TGF-β促癌效应来抑制人类肿瘤的恶化^[12]^。作为TGF-β对抗物，TGFBR3对于抑制肿瘤恶化具有潜在的治疗学意义。近几年来，研究者发现在许多肿瘤中存在TGFBR3的表达缺失情况，且表达降低或缺失与肿瘤的恶化程度相关。然而对TGFBR3表达缺失的研究并不全面，Finger等^[7]^研究发现在NSCLC中杂合子丢失可能是TGFBR3表达下调中的一种机制，但仅杂合子丢失未能完全阐明TGFBR3的表达缺失机制。已有报道^[10, 11]^指出在卵巢癌癌和前列腺癌肿中TGFBR3的表达可能受表观遗传调控，尽管如此，在NSCLC中*TGFBR3*基因启动子序列的CpG位点甲基化作用机制是否与TGFBR3的表达有关尚未见报道。我们推测*TGFBR3*基因的失活机理除基因缺失（杂合子丢失）和突变外，启动子区域的CpG岛甲基化也可能是该基因表达异常或失活的分子机制。在本实验研究中，我们利用Western blot检测TGFBR3在不同类型NSCLC细胞株和正常支气管上皮细胞中的表达情况，采用修饰DNA直接测序（bisulfite modification sequencing, BSP）对*TGFBR3*启动子区的CpG位点的甲基化状态进行分析，探讨*TGFBR3*基因表达失活的分子机制。

## 1 材料与方法

### 1.1 材料

#### 1.1.1 细胞株

NSCLC细胞系（A549、LTEP-a-2、SKMES-1、95D、SPC-A1、NCI-H460）购自中国科学院上海细胞所，正常支气管上皮细胞（human bronchial epithelial cell, HBEpiC）购自美国ATCC公司。

#### 1.1.2 主要试剂

RPMI-1640培养基为美国GIBCO公司产品，兔抗人TGFBR3多克隆抗体购自美国Abcam公司，小鼠抗人β-actin抗体及辣根过氧化物酶标记羊抗兔和羊抗鼠二抗均购自武汉博士德公司，CpGenome^TM^ Fast DNA Modification Kit购自Chemico公司。

### 1.2 方法

#### 1.2.1 细胞培养

采用10%的胎牛血清及含青、链霉素的RPMI-1640培养基培养A549、LTEP-a-2、SK-MES-1、95D、SPC-A1及NCI-H460细胞，HBEpiC应用推荐的培养基BECM进行培养，所有细胞置于37 ℃含5%CO_2_、95%空气的恒定湿度的细胞培养箱内进行培养。

#### 1.2.2 Western blot实验

RIPA裂解液裂解细胞，低温高速离心（4 ℃, 10 000 g）5 min后收集清液。BCA法测蛋白浓度，计算各样品蛋白浓度。每个细胞样取总蛋白50 μg，8%的SDS-聚丙烯酰胺凝胶电泳分离后，转印至PV DF膜（200 mA, 2 h），5%脱脂牛奶室温封闭2 h，加入一抗（兔抗人TGFBR3多克隆抗体，工作浓度1:1 000）、内参（β-actin抗体，1:1 000），4 ℃孵育过夜。之后加1:40 000稀释的HRP标记的羊抗兔二抗37 ℃孵育2 h。ECL试剂盒发光，暗室曝光胶片，扫描并分析条带的光密度值，应用SigmaScan Pro 5图像分析软件将图片上每个特异条带灰度值数字化。TGFBR3蛋白的灰度值除以内参基因产物β-actin蛋白的灰度值以校正误差，所得比值结果代表样品的TGFBR3蛋白相对含量，重复3次独立试验，取平均值。

#### 1.2.3 基因突变检测

##### 1.2.3.1 基因组DNA制备

参照QIAGEN DNeasy Tissue kit说明书提取细胞基因组DNA，紫外分光光度计鉴定，吸光度OD_260_/OD_280_≈1.8。

##### 1.2.3.2 PCR扩增

基因组DNA用于检测近端启动子区基本转录元件（-165到-75）的突变情况，上游引物：5′-CAC AGGCTCGAGCAGCATTC-3′，下游引物：5′-ATTACCCC CATCAGGCCGAC-3′。PCR反应体系为50 μL，50 ng-100 ng基因组DNA，1 unit Ex Taq DNA polymerase（Takara, Japan），0.2 μmol/L的上下游引物，1×Ex Taq Buffer（Mg^2+^ Plus），0.25 mol/L的dNTPs，加灭菌去离子水至50 μL。PCR反应参数：95 ℃预变性5 min；94 ℃变性30 s，61 ℃退火40 s，72 ℃延伸40 s为一个循环，共30次循环；最后一个循环后再72 ℃延伸10 min。扩增产物经2%琼脂糖凝胶电泳分析鉴定，纯化并测序（北京华大基因研究中心测序）。

#### 1.2.4 *TGFBR3*基因启动子区转录起始点上游序列分析

使用MethPrimer软件分析*TGFBR3*基因proximal promoter和distal promoter转录起始点上游约1 000 bp的核酸序列，预测TGFBR3基本转录元件所在区域CpG岛，并完成BSP（bisulfite sequence-PCR）的引物设计。

#### 1.2.5 重亚硫酸氢盐修饰及BSP检测

基因组DNA在进行甲基化分析前先用重硫酸氢钠盐进行修饰，修饰方法见CpGenome^TM^ DNA快速修饰试剂盒。简略概述如下：将1.0 μg的DNA溶于100 μL的双蒸水中，加入7 μL新配制的3 mol/L的NaOH溶液，于37 ℃变性10 min。然后加入550 μL新配制的DNA修饰剂（pH5.0），混匀。将混合物于55 ℃水浴20 h。加入750 μL的结合缓冲液后，用离心吸附柱纯化修饰后的DNA，然后加入50 μL 120 mmol/L NaOH/ 90%EtOH进行脱硫。最后，将DNA用30 μL-45 μL洗提缓冲液溶解，贮存于-20 ℃备用。取1 μL修饰后DNA作为模板，使用BSP引物（表 1）扩增，PCR循环条件为：95 ℃预变性5 min，94 ℃、30 s，多种退火温度45 s，72 ℃延伸1 min、15 s，35个循环，最后一个循环72 ℃延伸10 min。BSP产物电泳割胶回收纯化后送北京华大基因研究中心测序。

**1:** *TGFBR3*基因BSP引物 BSP primers used for *TGFBR3* gene

Region	Primer sequence 5 to 3'	Tm (℃)	Fragment size (bp)
Proximal promoter	FP: AAGGGAAGTATAGGTTYGAGTAG RP: ACTCTCACCTCCTACAAAAAAC	52	372
Distal promoter	FP: TTTTGAATTAAGAATTTTGTTTTTTT RP: AAACAAAAAAATCCCTTAAACC	53	114
Distal promoter	FP: GGGTTTAAGGGATTTTTTTG RP: ATTCTAACCAACATAATAAAACCCC	55	145
BSP: Bisulfite sequence-PCR.			

### 1.3 统计学处理

TGFBR3蛋白表达相对定量以β-actin作为内参照进行定量标定，应用TGFBR3与β-actin蛋白灰度值的比值作为相对表达量。数据以Mean±SD表示，7株细胞间TGFBR3的相对表达量的总体差异性比较采用单因素方差分析（*one*-*way* *ANOVA*），TGFBR3相对表达量在任意两株细胞间是否存在差异采用*bonferroni*检验。所有统计分析均使用SPSS 16.0统计软件进行运算，所有检验均为双侧检验，且以*P*＜0.05为具有统计学差异。

## 2 结果

### 2.1 TGFBR3在NSCLC细胞株和HBEpiC细胞中的表达

TGFBR3在6株NSCLC细胞和HBEpiC细胞中均有表达（图 1A），图 1B中展示了蛋白条带相对定量分析结果，以TGFBR3与β-actin蛋白灰度值的比值作为相对表达量。TGFBR3在7株细胞中的表达情况如表 2所示。以HBEpiC细胞中TGFBR3蛋白表达作为阳性对照，其蛋白表达相对量为0.48±0.02，而在6株NSCLC细胞株中TGFBR3蛋白的表达明显降低，分别为：SPC-A-1为0.11±0.02，LTEP-α-2为0.15±0.02，A549为0.14±0.01，NCI-H460为0.14 ±0.01，SK-MES-1为0.11±0.02，95D为0.08±0.01。6株NSCLC细胞与HBEpic细胞相比均存在统计学差异（*P*＜0.001），另外6株NSCLC细胞株中高转移株95D细胞与LTEP-α-2、A549、NCI-H460细胞相比TGFBR3蛋白表达也存在统计学差异（*P*值分别为0.003、0.017、0.011）（表 2）。

**1:**
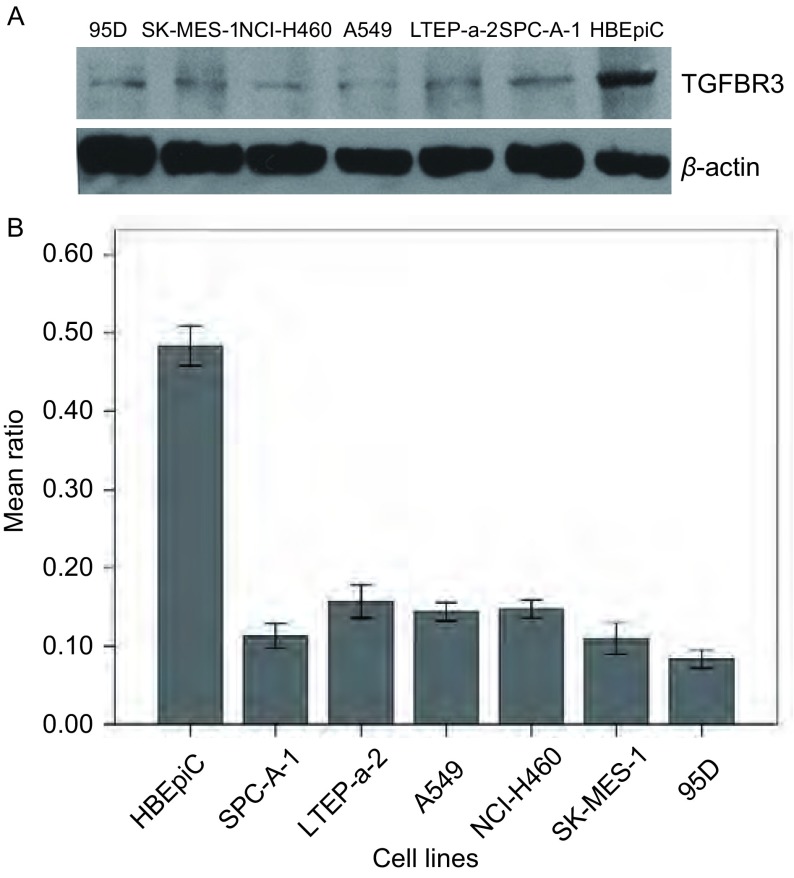
Western blot检测TGFBR3在NSCLC细胞及HBEpiC细胞中的表达情况。*β*-actin为内参照（A），及蛋白表达的相对定量结果（B）。 Expression levels of TGFBR3 were measured by Western blot in NSCLC cell lines and HBEpiC. A, *β*-actin used as an internal control; B, relative estimate for expression of TGFBR3.

**2:** TGFBR 3蛋白与*β*-actin在HBEpiC和6株NSCLC细胞株中的表达（Mean±SD） Expression levels of TGFBR3 and *β*-actin in HBEpiC and 6 NSCLC cell lines (Mean±SD)

Cell line	TGFBR3	*β*-actin	TGFBR3/*β*-actin	P^*^	P^**^
HBEpiC	127 757±6 670.91	263 272±832.46	0.48±0.02	--	< 0.001
SPC-A-1	42 377±5 521.98	368 743±3 590.93	0.11±0.02	< 0.001	1.00
LTEP-a-2	46 114±4 192.44	297 570±7 590.36	0.15±0.02	< 0.001	0.003
A549	41 051±2 902.01	290 789±3 317.54	0.14±0.01	< 0.001	0.017
NCI-H460	50 342±2 467.13	347 059±5 846.35	0.14±0.01	< 0.001	0.11
SK-MES-1	41 951±5 904.08	380 219±9 731.41	0.11±0.02	< 0.001	1.00
95D	37 538±3 812.74	442 607±11 509.24	0.08±0.01	< 0.001	--
P^*^: Difference in expression levels of TGFBR3 between HBEpiC and 6 NSCLC cell lines；P^**^: Difference in expression levels of TGFBR3 between 95D and other NSCLC cell lines.					

### 2.2 DNA序列分析

对6株NSCLC细胞株及HBEpiC proximal promoter区基本转录元件区（-165到-75）进行DNA测序发现7个样本DNA序列均无遗传突变。核酸序列（图 2）与Genbank中*TGFBR3*基因（GeneID: 7049）序列一致。

**2:**
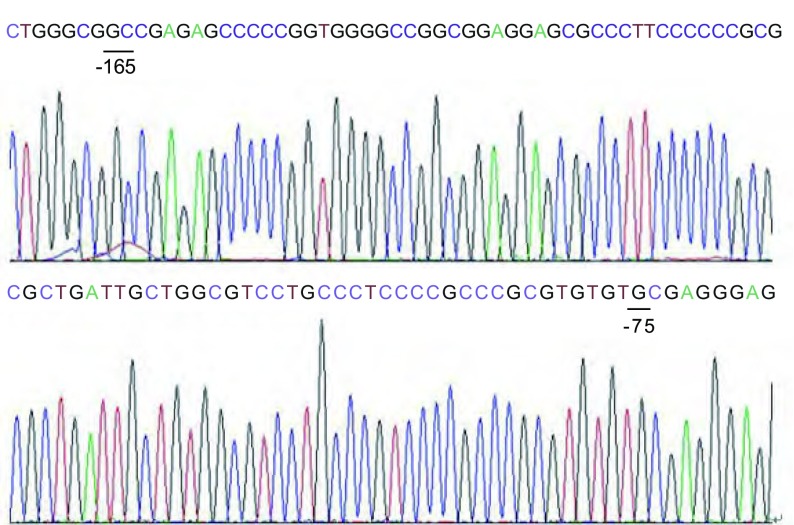
*TGFBR3*近端启动子区基本转录元件区（-165到-75）DNA测序结果 Schematic representative of DNA sequencing for the proximal promoter region from site -165 to -75 of *TGFBR3* gene

### 2.3 *TGFBR3*基因启动子区域序列特征及BSP测序结果

MethPrimer软件分析显示*TGFBR3*基因两启动子区转录起始点上游CpG含量丰富，均存在CpG岛（图 3，图 4）。Hempel等^[9, 10]^在卵巢癌（Ovca420）和乳腺癌（MDAMB-231）细胞株中应用荧光素酶报告分析法研究启动子活性发现*TGFBR3*基因proximal promoter中近转录起始点区域-165到-75处为活性最强的单位，另外在distal promoter中发现-314 bp到-199 bp区域表现为转录抑制作用。故此我们选取了*TGFBR3*基因-165到-75和-500 bp到-314 bp之间序列为检测区域。所研究的区域中转录起始位点上游-75到-165处共计出现13个CpG位点，分布在-82、-84、-88、-94、-103、-118、-120、-122、-134、-142、-145、-154、-164处，-314 bp到-199 bp区域我们检测8个CpG位点，分布在-292、-290、-286、-277、-270、-218、-214、-202处。BSP测序结果显示HBEpiC和6株NSCLC细胞株-165到-75区域13个CpG位点都未被甲基化（图 5），-314 bp到-199 bp区域8个位点均完全甲基化（图 6）。

**3:**
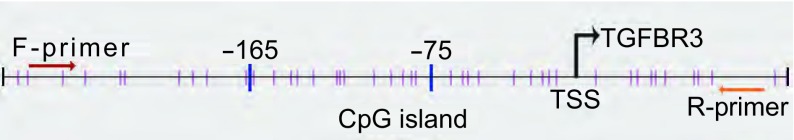
*TGFBR3*基因proximal promoter近转录起始位点处（-165到-75）CpG岛。TSS：转录起始位点。 A scheme of CpG islands covering the upstream sites from -165 to -75 of *TGFBR3* gene proximal promoter. TSS: transcription start site.

**4:**
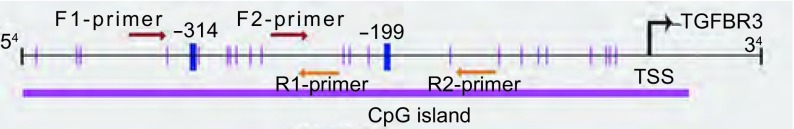
*TGFBR3*基因distal promoter区近转录起始位点处（-314到-199）CpG岛。TSS：代表转录起始位点。 A scheme of CpG islands covering the upstream sites -314 to -199 of *TGFBR3* gene distal promoter. TSS: transcription start site.

**5:**
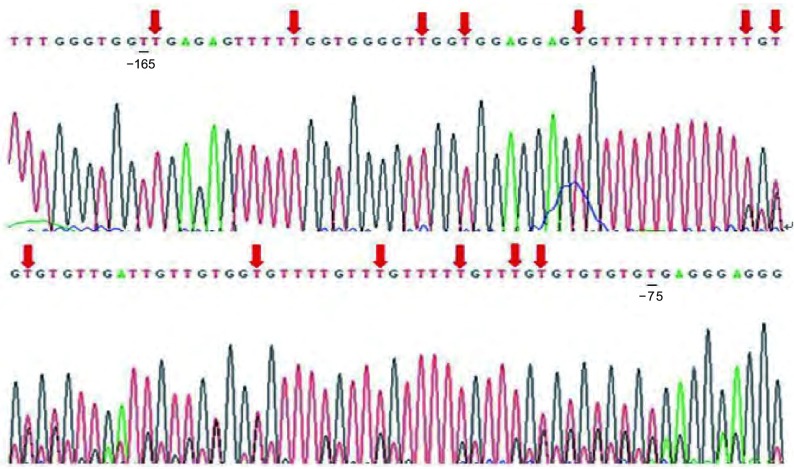
*TGFBR3*基因proximal promoter近转录起始位点处（-165到-75）BSP测序结果。红色箭头代表未甲基化CpG位点。 Schematic representative of BSP sequencing for the proximal promoter region from site -165 to -75 of *TGFBR3* gene. Red arrow, unmethylated CpG sites.

**6:**
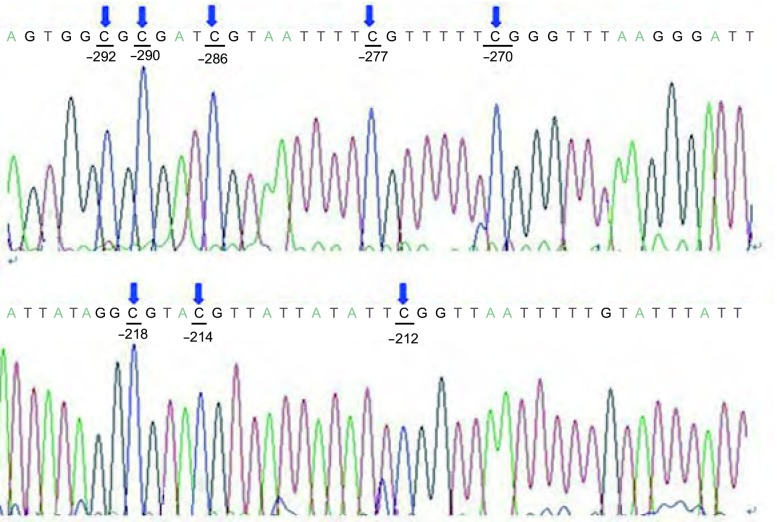
*TGFBR3*基因distal promoter区近转录起始位点处（-314到-199）BSP测序结果。蓝色箭头代表CpG甲基化位点。 Schematic representative for BSP sequencing for the proximal promoter region from site -314 to -199 of *TGFBR3* gene. Blue arrow, methylated CpG sites.

## 3 讨论

TGF-β是一类多功能的多肽类生长因子，在调控细胞内环境稳定、胚胎发育、细胞增殖、分化、转移、凋亡和免疫监控过程中发挥重要作用，与人类多种疾病尤其是肿瘤的发生发展密切相关。它通过与其表面特异性受体结合组成TGF-β信号通路发挥细胞学效应。目前发现3种TGF-β特异性受体：Ⅰ型（TGFBR1）、Ⅱ型（TGFBR2）和Ⅲ型（TGFBR3），其Ⅰ、Ⅱ型受体是信息传递分子，介导TGF-β信号通路发挥细胞学效应，Ⅲ型受体不同于Ⅰ、Ⅱ型受体，它没有激酶功能域，未发现其直接参与信息传递，在功能上具有二元性^[13]^：一方面为TGF-β的一种特异性辅助受体，促进TGF-β信号通路的进行；另一方面作为抑癌基因，在多种肿瘤中能抑制肿瘤细胞的侵袭、转移和血管发生作用，负性调控癌症进程。

大量证据显示*TGFBR3*作为抑癌基因，在许多人类癌症中存在表达缺失或降低情况。与TGF-β信号通路其它组分相比，TGFBR3的表达降低或缺失是一个更为频繁的事件^[15-17]^。TGFBR3的表达降低常伴随着成神经细胞瘤^[17]^、卵巢癌^[10, 18]^、卵巢粒层细胞瘤^[19]^、子宫内膜癌^[20]^、前列腺癌^[11, 14]^、肾细胞癌^[21]^、乳腺癌^[9]^及胰腺癌^[16, 22]^等肿瘤恶化程度的增加。本研究检测到与正常支气管上皮细胞相比，NSCLC细胞系中TGFBR3蛋白表达明显下凋，这与Finger等^[7]^在NSCLC组织中检测到的结果类似，表明TGFBR3对NSCLC的发生发展起到重要的作用。另外，6株NSCLC细胞株中，高转移株NSCLC细胞95D与其它3种非转移NSCLC细胞（LTEP-α-2、A549、NCI-H460）相比，TGFBR3蛋白表达水平显著降低，提示在NSCLC中TGFBR3与细胞转移潜能相关，TGFBR3可能影响肿瘤细胞的侵袭和转移作用。研究结果从细胞水平上进一步证实了TGFBR3的表达下调或缺失与NSCLC癌症进程呈负性相关。

关于肿瘤进程中TGFBR3的表达下调或缺失的机制目前存在若干种解释。*TGFBR3*基因位于染色体1p33-p32区域内，这个区域在许多人类肿瘤包括乳腺癌、胃癌、结肠直肠癌、子宫内膜癌、肾癌、肺癌、卵巢癌中频繁缺失^[23]^。在乳腺癌、前列腺癌、NSCLC和肝癌中发现20%-50%患者*TGFBR3*基因发生杂合性丢失（loss of heterozygosity, LOH）^[9, 11, 13, 16]^，但在多数肿瘤样本中，TGFBR3的低表达或缺失并不与LOH相匹配，提示存在其它机制负性调控TGFBR3的表达。*TGFBR3*基因proximal promoter中近转录起始点区域-165到-75区为活性最强的单位，该区域包含SP1结合位点，该片段的缺失或变异会导致TGFBR3表达水平的明显下降，鉴于此，我们对该区域进行了突变检测，结果显示6株NSCLC和HBEpiC中均无任何碱基突变，这与Bae等^[15]^在肝癌样本中的研究结果相类似。此外，有个别研究^[24]^报道了*TGFBR3*基因5′UTR区、3′UTR区和ORF区的存在单核苷酸多肽性（single nucleotide polymorphism, SNP），但并未发现这些SNP具有生物学效应。Hempel等^[8]^在卵巢癌细胞株中发现使用甲基转移酶抑制剂和组蛋白去乙酰化酶抑制剂（TSA）处理能提高*TGFBR3*基因mRNA水平上的表达，提示在此类肿瘤中表观遗传调控机制可能是导致其表达水平下降的潜在机制。然而，关于*TGFBR3*基因异常表达的表观遗传调控机制尚未得到深入研究。

抑癌基因的失活通常由遗传和表观遗传机制造成，遗传缺失和点突变能使基因表达异常，而表观遗传修饰也可能导致基因表达异常。人类DNA的表观遗传修饰的主要形式是甲基化修饰，并且许多抑癌基因原本非甲基化的CpG岛的超甲基化与基因的表达失活相关。目前国内外对TGFBR3缺失机制的研究较少，而关于其CpG岛甲基化状态和基因表达水平与NSCLC发生的关系迄今为止还未在表观遗传调节方面做阐述。为了在NSCLC细胞中阐明可能会影响TGFBR3表达的表观遗传机制，我们分析研究了*TGFBR3*基因的甲基化情况，选取该基因proximal promoter和distal promoter序列进行了生物信息学分析。

分析结果显示，这两个启动子区序列均存在CpG岛，有高甲基化或低甲基化的基础。且Hempel等研究提示DNA甲基化修饰很可能参与了该基因的表达调控，作为基本转录元件的proximal promoter-165到-75区和作为重要转录抑制元件的distal promoter-314到-199区的甲基化状态可能会与TGFBR3表达相关。本实验应用BSP法对*TGFBR3*基因的预测启动子序列进行甲基化分析。测序结果显示，正常表达*TGFBR3*基因的HBEpiC及表达下调的NSCLC细胞株其启动子区-165到-75序列均无甲基化，研究结果提示，*TGFBR3*基因启动子区重要转录元件-165到-75区域的甲基化发生状态并不是*TGFBR3*基因表达下调的主要原因，但是在其它肿瘤细胞中还没有此类报道，我们不排除在其它肿瘤中该部位发生甲基化的可能性。Distal promoter-314到-190区8个CpG位点在所有细胞株中都完全甲基化，表明该区域的甲基化状态与TGFBR3表达缺陷并无相关性。另外我们在随机抽取的几例NSCLC组织及相应癌旁组织样本中发现了同样的情况。我们的研究结果提示*TGFBR3*基因启动子区重要转录元件的甲基化异常现象可能在NSCLC发生发展过程中并不是一个常见事件。关于*TGFBR3*基因失活的原因还需进一步探讨。然而，我们的研究为全面阐述NSCLC的DNA甲基化谱提供了研究数据。
